# Microtubules in Microorganisms: How Tubulin Isotypes Contribute to Diverse Cytoskeletal Functions

**DOI:** 10.3389/fcell.2022.913809

**Published:** 2022-07-05

**Authors:** Abesh Bera, Mohan L. Gupta

**Affiliations:** Genetics, Development, and Cell Biology, Iowa State University, Ames, IA, United States

**Keywords:** tubulin isotype, tubulin, microtubule, microorganism, cytoskeleton

## Abstract

The cellular functions of the microtubule (MT) cytoskeleton range from relatively simple to amazingly complex. Assembled from tubulin, a heterodimeric protein with α- and β-tubulin subunits, microtubules are long, hollow cylindrical filaments with inherent polarity. They are intrinsically dynamic polymers that utilize GTP binding by tubulin, and subsequent hydrolysis, to drive spontaneous assembly and disassembly. Early studies indicated that cellular MTs are composed of multiple variants, or isotypes, of α- and β-tubulins, and that these multi-isotype polymers are further diversified by a range of posttranslational modifications (PTMs) to tubulin. These findings support the multi-tubulin hypothesis whereby individual, or combinations of tubulin isotypes possess unique properties needed to support diverse MT structures and/or cellular processes. Beginning 40 years ago researchers have sought to address this hypothesis, and the role of tubulin isotypes, by exploiting experimentally accessible, genetically tractable and functionally conserved model systems. Among these systems, important insights have been gained from eukaryotic microbial models. In this review, we illustrate how using microorganisms yielded among the earliest evidence that tubulin isotypes harbor distinct properties, as well as recent insights as to how they facilitate specific cellular processes. Ongoing and future research in microorganisms will likely continue to reveal basic mechanisms for how tubulin isotypes facilitate MT functions, along with valuable perspectives on how they mediate the range of conserved and diverse processes observed across eukaryotic microbes.

## Introduction

**FIGURE 1:**
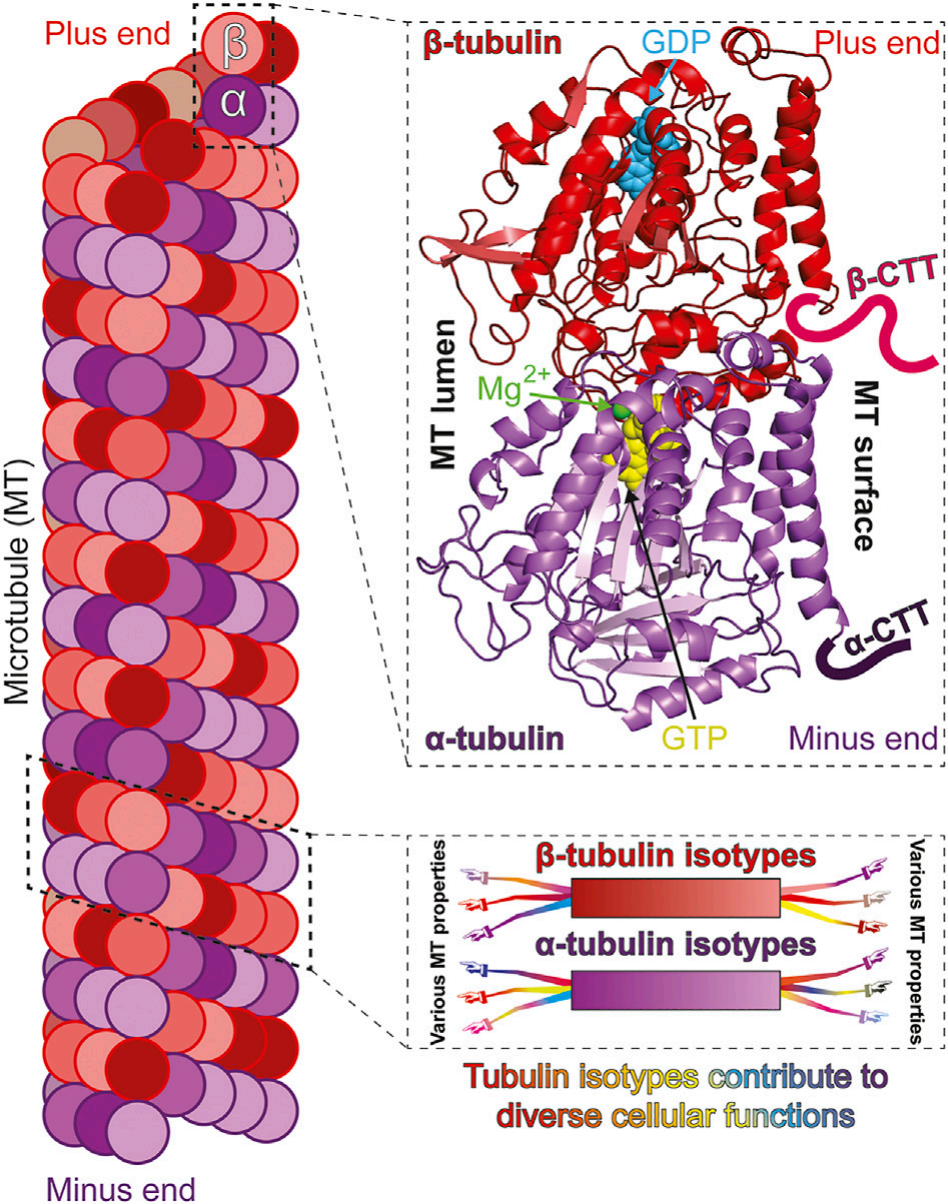
Isotypes of α- and β-tubulin construct functionally diverse microtubules. Microtubules (MTs) polymerize from “head-to-tail” binding of tubulin, a heterodimer of α- and β-tubulin subunits (left). Both α- and β-tubulin subunits bind GTP but only the β-tubulin bound GTP is hydrolysable and exchangeable [top-right; cryo-EM structure of *S. cerevisiae* tubulin, polymerized with GTP in vitro; PDB ID: 5W3F (Howes et al., 2017)]. The carboxy terminal tails (CTTs) are normally unstructured and are only drawn representatively and not to scale. The α- and β-tubulin subunits can be encoded by multiple genes to produce variants, or isotypes (bottom-right; color gradient represents different isotypes). Tubulin isotypes can each possess common and/or unique molecular properties which expands the ability of MTs to optimally perform diverse and specialized functions (represented by arms/hands differentially extending from isotypes).

Microtubules (MTs) are essential, intrinsically dynamic cytoskeletal filaments, polymerized from the heterodimeric protein tubulin. Each heterodimer contains two closely related, GTP-binding subunits: α-tubulin and β-tubulin (Figure 1). Populations of MTs in eukaryotes mediate critical functions like cell division, cell migration, and intracellular cargo transport. These processes require a high degree of fidelity to ensure cell viability, genome stability and organismal health. Thus, MTs must support a diverse range of interactions and their nucleation, dynamics and stability must be tightly regulated in both space and time.

In addition to α- and β-tubulin, the protein family also includes the ubiquitous mediator of MT nucleation, γ-tubulin (Oakley et al., 2015) and the specialized δ-, ε- and ζ-tubulins that are present in a subset of eukaryotes in which they facilitate the structure and/or function of centrioles and basal bodies (Chang and Stearns, 2000; Vaughan et al., 2000; Turk et al., 2015). Some organisms harbor two or three variants, or isotypes, of γ-tubulin (Findeisen et al., 2014). Notably, almost all organisms utilize multiple isotypes of α- and β-tubulin to build tubulin heterodimers (Cleveland et al., 1980; Roll-Mecak, 2019). The functional role of these tubulin isotypes, however, remains largely obscure.

The β- and α-subunits of two consecutive tubulin heterodimers interact to polymerize into inherently polarized protofilaments. Lateral interactions between typically 13 protofilaments yield the hollow cylindrical structure of MTs (Figure 1). While both α- and β-tubulin bind GTP, nucleotide binding to α-tubulin appears mainly structural while MT assembly and disassembly is driven by the binding and subsequent hydrolysis, respectively, of GTP bound to the β-subunit (Figure 1) [reviewed in (Nogales, 2001)]. The transition between MT assembly and disassembly is stochastic and the phenomenon termed “dynamic instability” (Mitchison and Kirschner, 1984). The inherent polarity and dynamic instability of MTs facilitates kinesin/dynein mediated directed cargo transport (Barlan and Gelfand, 2017), MT organization (Mani et al., 2021), MT Associated Protein (MAP) interaction (Brouhard and Rice, 2018), as well as MT-dependent force generation (Forth and Kapoor, 2017).

MT behavior is controlled at multiple levels. They are regulated at the level of MT nucleation by γ-tubulin, at the level of fundamental polymer dynamics by α/β heterodimers, and at the level of macromolecular organization by a range of MT Associated Proteins (MAPs) [reviewed in (Goodson and Jonasson, 2018)]. Another layer of regulation comes from a relatively large number of posttranslational modifications (PTMs) that occur on tubulin [reviewed in (Janke and Magiera, 2020)]. A third aspect of MT regulation stems from the multiple tubulin variants, or isotypes, expressed in most cells (Figure 1) (Nsamba and Gupta, 2022). Relative to our understanding of how PTMs and MAPs influence MT function, which can be significant, our knowledge of how tubulin isotypes themselves contribute to the complexity of MT behaviors remains limited. Despite the contribution of PTMs and MAPs, in this review we mainly focus on the evidence that tubulin isotypes play a fundamental role in the diversity of MT functions.

Why organisms express multiple α- and β-tubulin isotypes has been a central, long-standing question. The multi-tubulin hypothesis, formulated decades ago, postulates that specific tubulin isotypes contribute unique functional properties to MTs (Figure 1) (Cleveland, 1987; Ludueña, 1993). The discovery that mutations in certain mammalian tubulin isotypes cause specific disorders, collectively known as tubulinopathies, lends support to this idea and emphasizes the need to elucidate the cellular roles of various isotypes (Bahi-Buisson and Maillard, 2016; Romaniello et al., 2018; Binarová and Tuszynski, 2019). Directly testing the multi-tubulin hypothesis, however, has been hampered by significant challenges. Depletion of one may lower overall tubulin levels and/or alter the relative ratios of the remaining isotypes. Excess β-tubulin relative to α-subunits can be toxic (Weinstein and Solomon, 1990), and overexpression can lead to aggregated, non-functional protein (Bhattacharya and Cabral, 2004). Thus, interpretation of cell-based experiments has often been confounded by these indirect effects. Additionally, the purification of mutant and/or single isotype tubulin was essentially restricted to yeast (Kilmartin, 1981; Davis et al., 1994; Bode et al., 2003; Drummond et al., 2011), and only recently obtained from higher eukaryotes (Minoura et al., 2013; Pamula et al., 2016; Ti et al., 2016; Vemu et al., 2017; Ayukawa et al., 2021). Thus, directly testing the role of tubulin isotypes in the multi-tubulin hypothesis has been challenging. Eukaryotic microorganisms served as pioneering model systems to investigate the function of tubulin isotypes. These organisms offer the tractability and accessibility needed to address the question. While advances in genome editing and recombinant tubulin purification have alleviated some of the constraints and challenges in more complex, higher eukaryotes, microorganisms continue to offer advantages for elucidating the fundamental mechanisms of tubulin isotypes. Microorganisms harbor conserved MT structures, governed by homologous regulators (PTMs and MAPs), that perform functions analogous to their higher eukaryotic counterparts. They are highly tractable, allowing ease of genetic manipulation, quantification of MT-dependent processes, and in some, continuous monitoring of individual MTs during these processes. Some use relatively fewer tubulin isotypes and offer a simplified landscape (Table 1) (Toda et al., 1984; Schatz et al., 1986a) while many, like their higher eukaryotic counterparts, employ many isotypes despite their unicellular status (Suprenant et al., 1985; Eisen et al., 2006). Moreover, some microorganisms such as budding yeast do not utilize any or very few PTMs, further simplifying the interpretation of tubulin isotype manipulation. Indeed, the study of single isotypes, expressed at levels comparable to total tubulin isotypes in normal cells, has only been achieved using microorganisms (Nsamba et al., 2021).

**TABLE 1:** Multiple tubulin isotypes are expressed in model microorganisms*

Model microorganism	Division/Phylum	Tubulin isotypes/subunit				References
		alpha-tubulin genes		beta-tubulin genes		
*Saccharomyces cerevisiae*	Ascomycota	2	*TUB1, TUB3*	1	*TUB2*	Neff et al. (1983), Schatz et al. (1986a), Schatz et al. (1986b)
*Schizosaccharomyces pombe*	Ascomycota	2	*nda2, atb2*	1	*nda3*	Hiraoka et al. (1984), Toda et al. (1984)
*Tetrahymena thermophilus*	Ciliophora	4	*ATU1, ALT1-3*	8	*BTU1, BTU2, BLT1-6*	Callahan et al. (1984), Suprenant et al. (1985), Gaertig et al. (1993), Eisen et al. (2006)
*Aspergillus nidulans*	Ascomycota	2	*tubA, tubB*	2	*benA, tubC*	Sheir-Neiss et al. (1978), Morris et al. (1979), Morris et al. (1984), May et al. (1987); Doshi et al. (1991)
*Fusarium graminearum*	Ascomycota	2	*FgTUB4, FgTUB5*	2	*FgTUB1, FgTUB2*	Lu et al. (2000), Zhao et al. (2014)
*Chlamydomonas reinhardtii*	Chlorophyta	2	*tua1, tua2*	2	*tub1, tub2*	Minami et al. (1981), Silflow and Rosenbaum (1981), Brunke et al. (1982b), Youngblom et al. (1984); James et al. (1993)
*Physarum polycephalum*	Amoebozoa	5	*altA, altB(N), altB(E), altC, altD*	3	*betA, betB, betC*	Burland et al. (1983), Burland et al. (1984), Burland et al. (1988); Schedl et al. (1984b), Krämmer et al. (1985); Singhofer-Wowra et al. (1986), Monteiro and Cox (1987a), Monteiro and Cox (1987b), Green et al. (1987), Singhofer-Wowra and Little (1987), Solnica-Krezel et al. (1988), Werenskiold et al. (1988)
*Toxoplasma gondii*	Apicomplexa	3	α_1_, α_2,_ α_3_	3	β_1_, β_2_, β_3_	Nagel and Boothroyd (1988), Hu et al. (2006), www.toxodb.org

*In Table 1, gene names are presented using organism-specific syntax.

Microorganisms played a critical role in the discovery of tubulin family genes and the initial characterizations of α and β tubulin isotypes. Genes encoding the tubulin family members γ (Oakley and Oakley, 1989), δ (Dutcher and Trabuco, 1998; Fromherz et al., 2004) and ε-tubulin (Goodenough and StClair, 1975; Dutcher et al., 2002) were first identified by exploiting model microorganisms. The latter two were also shown to be critical for cleavage furrow placement in *Chlamydomonas*. Moreover, beginning about 40 years ago it was recognized that most microorganisms harbor multiple isotypes of α and/or β tubulin. The tractability of model microorganisms facilitated pioneering research on tubulin isotypes. Although these early studies indicated that isotypes make distinct contributions to MT function, they also revealed relatively large redundancies among isotypes for basic MT processes. This redundancy, together with the technical limitations at that time, worked to dampened deeper exploration into tubulin isotype function in many models. In the last decade, however, a flurry of research in certain models like budding yeast, fission yeast and Tetrahymena, has yielded significant insights into the function of tubulin isotypes. The role of PTMs in regulating MT function is also becoming increasingly clear and has been reviewed recently (Wloga et al., 2017; Janke and Magiera, 2020). In this review we focus on what has been learned about the function of α- and β-tubulin isotypes using model microorganisms (Tables 1, 2). We emphasize the trajectory of progress in budding yeast*,* as arguably the most studied system, along with what has been found in fission yeast and Tetrahymena. We summarize what is known in a range of other models to highlight the common aspects of significant redundancy coupled with unique contributions of tubulin isotypes, and the continuing opportunity to exploit these systems to learn how they facilitate the wide diversity of MT-dependent processes observed across biology. Together microorganisms hold significant potential to elucidate conserved, fundamental mechanisms by which tubulin isotypes underlie MT properties and function.

**TABLE 2:** Microbial tubulin isotypes optimize and mediate differential organismal and cellular functions^*^.

Model microorganism	Tubulin isotypes possessing distinct functions		Functional significance of microbial tubulin isotypes
	alpha-tubulin genes	beta-tubulin genes	
*Saccharomyces cerevisiae*	*TUB1, TUB3*		***TUB1*:** Mitosis, meiosis, conjugation, benomyl hyper-resistance Schatz et al. (1986a); Schatz et al. (1986b), high MT dynamicity *in vitro* and *in vivo* Bode et al. (2003), Nsamba et al. (2021), Dyn1 mediated spindle positioning, Dyn1 pathway MAP localization Nsamba et al. (2021); ***TUB3*:** Benomyl hyper-sensitivity Nsamba et al. (2021), low MT dynamicity *in vitro* and *in vivo* Bode et al. (2003), Nsamba et al. (2021), Kar9 mediated spindle positioning, Kar9 pathway MAP localization Denarier et al. (2021); Nsamba et al. (2021).
*Schizosaccharomyces pombe*	*nda2, atb2*		***nda2*:** Karyogamy, nuclear positioning, duplicated centrosome migration, thiabendazole sensitivity Toda et al. (1981), Toda et al. (1983); Adachi et al. (1986), slower apparent rate constants for MT assembly, slower growth rate and lower critical concentration of MT polymerization *in vitro* Hussmann et al. (2016); ***atb2*:** spindle assembly checkpoint, heterodimer assembly Radcliffe et al. (1998), interphase MT dynamics, EB1/Mal3 localization Asakawa et al. (2006).
*Tetrahymena thermophila*	*ATU1,*	*BTU1, BTU2, BLT1, BLT4*	***ATU1*:** Cell viability Hai et al. (1999), ciliary and cytoplasmic MT generation Gu et al. (1995); ***BTU1*:** Cell viability Xia et al. (2000), ciliary and cytoplasmic MT generation Gu et al. (1995); ***BTU2*:** Cell viability Xia et al. (2000), ciliary MT generation Gu et al. (1995), enriched in somatic cilia and basal bodies Pucciarelli et al. (2012); ***BLT1*:** Macronuclear MT and spindle assembly, micronuclear meiotic spindle assembly during conjugation Pucciarelli et al. (2012); ***BLT4*:** Macronuclear MT and spindle assembly Pucciarelli et al. (2012).
*Aspergillus nidulans*	*tubA, tubB*	*benA, tubC*	***tubA*:** MT stability Gambino et al. (1984), karyokinesis, cell/nuclear morphology Doshi et al. (1991), asexual vegetative growth Oakley et al. (1987), Doshi et al. (1991), sexual development before the first meiotic division Kirk and Morris (1991); ***tubB*:** karyokinesis, cell/nuclear morphology Doshi et al. (1991); ***benA*:** MT instability Gambino et al. (1984), nuclear migration and karyogamy during vegetative growth Oakley and Morris (1980), Oakley and Morris (1981); Oakley and Rinehart (1985), benomyl hyper-sensitivity May (1989); ***tubC*:** conidiation May and Morris (1988), MT function during conidiation May et al. (1985), benomyl hyper-sensitivity May (1989).
*Fusarium graminearum*		*FgTUB1, FgTUB2*	***FgTUB1*:** Benzimidazole sensitive Chen et al. (2007), perithecia formation and sexual development Zhao et al. (2014), ascosporogenesis Zhao et al. (2014), Wang et al. (2019); ***FgTUB2*:** Benzimidazole resistance Chen et al. (2009), vegetative development Zhao et al. (2014), mycotoxin synthesis Wang et al. (2019).
*Chlamydomonas reinhardtii*	*tua1, tua2*	*tub1, tub2*	**each isoform:** differential anti-tubulin drug resistance Kato-Minoura et al. (2020).
*Physarum polycephalum*	*altA, altB(N), altB(E),*	*betA, betB, betC*	***altA, altB(N), altB(E)*:** MT assembly in plasmodia and myxamoeba Burland et al. (1983), Roobol et al. (1984), Cunningham et al. (1993); Gerber et al. (2022), *altA* builds flagellar MTs via PTM in amoeba and flagellates Green and Dove (1984), Blindt et al. (1986), Diggins and Dove (1987), Sasse et al. (1987); ***betA*:** MT structure assembly during myxamoeba stage Burland et al. (1988), Diggins-Gilicinski et al. (1989), Gerber et al. (2022); ***betB*:** constitutively expressed in plasmodia and myxamoeba Werenskiold et al. (1988), Paul et al. (1989), benzimidazole resistance to both plasmodia and myxamoeba Burland et al. (1984); ***betC*:** anastral mitotic spindle assembly in plasmodia Burland et al. (1983), Roobol et al. (1984), Gerber et al. (2022), MT assembly in developing cells during amoeboid-plasmodial transition Solnica-Krezel et al. (1988).
*Toxoplasma gondii*	α_1_, α_2,_ α_3_	β_1_, β_2_, β_3_	**α**_**1**_ **, β**_**1**_ **, β**_**2**_ **:** compose cortical MTs Wang et al. (2021), α_1_ demonstrates dinitroaniline resistance Morrissette et al. (2004), Ma et al. (2007), Ma et al. (2010); **α**_**2,**_ **α**_**3**_ **:** potentially unique role(s) in conoid and flagellar axonemal MTs Morrissette (2015).

*In Table 2, gene names are presented using organism-specific syntax.

### Saccharomyces cerevisiae

The initial purification of budding yeast tubulin in 1981 provided evidence, *via* 2-D gel electrophoresis, that the yeast cytoskeleton utilizes multiple tubulin isotypes (Kilmartin, 1981). Within 5 years, the full complement of one β-tubulin, *TUB2* (Neff et al., 1983), and two α-tubulin isotypes, *TUB1* and *TUB3* (Schatz et al., 1986a)*,* was identified (Table 1). While the single β-tubulin is essential, the α-isotype genes are not equivalent in their ability to support viability. Cells survive disruption of *TUB3* but not *TUB1* (Schatz et al., 1986b). Northern blots with either gene as probe suggested that *TUB1* transcript was more prevalent than *TUB3*, although probe cross-hybridization prevented accurate quantification (Schatz et al., 1986a). The idea that cells harbor more Tub1 than Tub3 subunits is consistent with the finding that although lethal, *tub1∆* can be rescued by increased expression of *TUB3* (Schatz et al., 1986b)*.* Yet, while *tub3∆* cells can undergo mitosis, conjugation and meiosis, they display decreased spore viability and hypersensitivity to the microtubule destabilizer benomyl (Schatz et al., 1986b). Increased *TUB1* expression in *tub3∆* cells rescues benomyl sensitivity, however, the sensitivity of *tub3∆* cells made viable by additional *TUB3* was not determined (Schatz et al., 1986b). Combining the results of these studies, and the fact that *TUB1* and *TUB3* share 90% amino acid identity, but only ∼73% with α-tubulins from porcine and fission yeast, it was proposed that they are functionally redundant, with the observed phenotypic effects due to relative expression levels (Schatz et al., 1986a). Subsequent studies supported the observation of more Tub1 relative to Tub3 in soluble protein extracts and purified tubulin (Bode et al., 2003; Hanson et al., 2016; Aiken et al., 2018), but it would be 35 years before a more detailed analysis of Tub1 and Tub3 function *in vivo* was reported (Nsamba et al., 2021).

The yeast model has been exploited to investigate the regulation of cellular tubulin levels. It was initially shown that Tub1 (α-tubulin) and Tub2 (β-tubulin) polypeptide levels do not increase proportionally with gene copy number. In cells harboring extra copies of either *TUB1* or *TUB1* plus *TUB2*, expression was found to be downregulated at both the transcript and polypeptide levels, such that cells contained near normal levels of Tub1, Tub2 and Tub3 protein (Katz et al., 1990). In contrast to downregulation, budding yeast appear incapable of upregulating tubulin expression when needed. In diploid *TUB2*/*tub2∆* hemizygous cells, Tub2 protein levels are approximately half that seen in normal *TUB2*/*TUB2* cells. Moreover, both Tub1 and Tub3 α-tubulin protein levels were concomitantly reduced by 50% (Katz et al., 1990). Similarly, total α-tubulin levels are reduced in *tub3∆* cells (Hanson et al., 2016; Aiken et al., 2018; Denarier et al., 2021; Nsamba et al., 2021), and although *tub1∆* cells are inviable, they are rescued by increased *TUB3* expression (Schatz et al., 1986b).

Methods to purify native (Barnes et al., 1992) and recombinant (Davis et al., 1994) yeast tubulin, including use of affinity tags (Gupta et al., 2002) and overexpression (Johnson et al., 2011) have yielded critical insights into areas of tubulin structure-function (Gupta et al., 2002; Ayaz et al., 2012), modulation of microtubule dynamics by GTP hydrolysis (Davis et al., 1994), and the dynamic properties of each isotype in reconstitution assays (Bode et al., 2003). Relative to Tub3, microtubules polymerized entirely with Tub1 display increased catastrophe frequency and depolymerization rate, leading to overall higher dynamicity. Microtubules containing a mixture of Tub1 and Tub3 α-subunits have properties intermediate to those made exclusively from Tub1 or Tub3. Thus, the benomyl sensitivity seen in *tub3∆* cells could arise, at least in part, from the decreased stability of exclusively Tub1 microtubules. SDS-PAGE also showed that mixtures purified from wildtype cells contain ∼80% Tub1 and 20% Tub3 (Bode et al., 2003). Consistent with this observation, western blots of soluble cell lysates from *tub3∆* cells have ∼70% of wildtype α-tubulin levels (Hanson et al., 2016), while those from wildtype cells show ∼70% Tub1 and 30% Tub3 (Aiken et al., 2018).

With the advent of Green Fluorescent Protein, independent groups concurrently showed that GFP-Tub1 (Straight et al., 1997) and GFP-Tub3 (Carminati and Stearns, 1997) both incorporate into all microtubule structures during vegetative growth. As in other organisms, this technical advance greatly facilitated the elucidation of mechanisms controlling microtubule regulation and organization. At the tubulin molecular level, significant attention has focused on the highly variable carboxy-terminal tail (CTT). The CTT of α-tubulin is important for + TIP protein binding to microtubules and dynein-mediated nuclear movements (Badin-Larçon et al., 2004). It is also involved in membrane-bound cargo trafficking (Boscheron et al., 2016). On β-tubulin, the CTT is a critical mediator of kinesin-5 interaction (Aiken et al., 2014) and needed for accurate chromosome segregation (Fees et al., 2016). Considering these findings and the unique synthetic genetic interactions identified for either α-isotype (Nsamba et al., 2021) it is possible the CTTs of Tub1 and Tub3 possess isotype-specific properties.

Two recent studies address the function of Tub3 *in vivo*. Both examined Tub1 level in *tub3∆* cells and found it to be between 37%–64% (Denarier et al., 2021) and ∼50% (Nsamba et al., 2021) of the total α-tubulin level in wildtype cells. Consistent with these results, the *TUB1* and *TUB3* transcript levels are equivalent in wildtype cells, and *TUB1* mRNA level unchanged in *tub3∆* cells (Nsamba et al., 2021). Quantitative imaging of mRUBY-Tub1 and mRUBY2-Tub3 also indicates that they constitute equal proportions of the α-tubulin pool (Denarier et al., 2021). At present, the discrepancy in Tub1 to Tub3 ratios between these results and previous studies remains unclear. One explanation for more Tub1 in purified mixtures and prior immunoblot analyses could be that Tub1-containing tubulin heterodimers are more successfully extracted in non-denaturing buffers and/or more tolerant of purification procedures compared to those containing Tub3. Another, more interesting possibility is that the isotypes may be independently regulated under the conditions used for the various experiments.

Cells respond differently to high overexpression of Tub1 or Tub3. Whereas expressing *TUB1* from the strong GAL1 promoter is well tolerated (Burke et al., 1989), expressing *TUB3* from the strong TEF1 promoter results in tiny colonies with sickly cells (Denarier et al., 2021). Notably, cells overexpressing *TUB3* as their sole source of α-tubulin display nearly double the typical number of microtubules in pre-anaphase spindles, emanating from significantly enlarged spindle pole bodies (Denarier et al., 2021). The increased SPB diameter is larger than would be predicated for the somewhat slower spindle assembly in these cells (Denarier et al., 2021), and thus may be a direct consequence of the increased Tub3. Additionally, localization of the negative dynein regulator, She1, to spindles containing exclusively Tub3 is increased 62% compared to spindles in wildtype cells (Denarier et al., 2021). She1 also shows increased localization, albeit slightly, to Tub3 compared to Tub1 microtubules *in vitro* (Denarier et al., 2021). Thus, the highly increased localization seen *in vivo* could be indicative of Tub3-specific mechanisms, but may also result from more microtubules present in the Tub3 spindles (Denarier et al., 2021).

**FIGURE 2:**
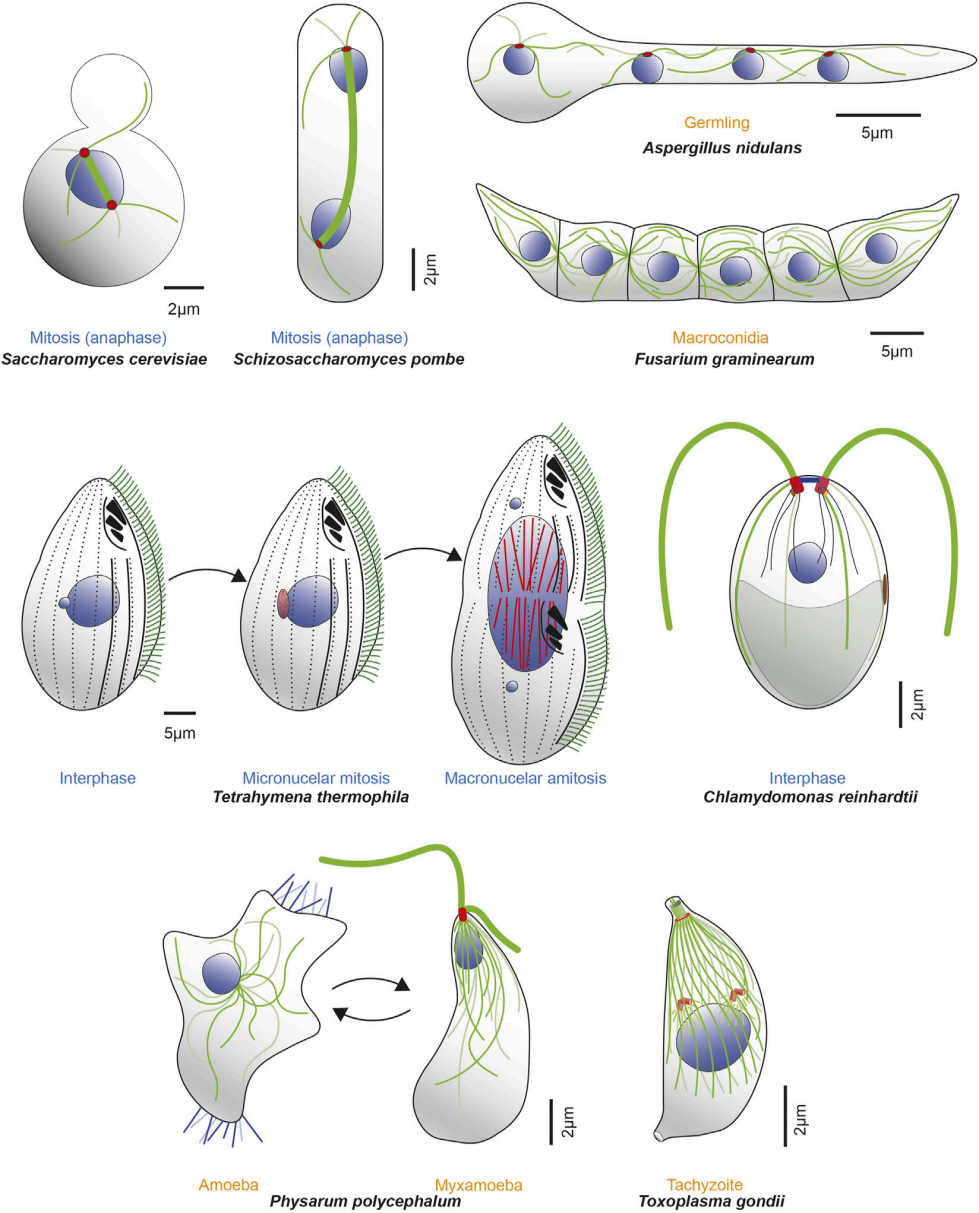
Microorganisms express one or more α- and β-tubulin isotypes to construct various MT-organelles and to perform diverse functions with a range of complexity. Example microorganisms are represented in either different cell cycle (blue text) or life cycle (orange text) stages, along with MTs (dark and light green lines), mitotic spindle (thick green line), nuclei (blue) and corresponding MTOCs (red). Top row (from left to right, clockwise): schematics of mitotic *S. cerevisiae* in anaphase; mitotic *S. pombe* in anaphase; tetranucleate, interphase *Aspergillus nidulans* germling (germinated from conidia, an asexual, uninucleate spore produced during vegetative life cycle. Septa are not formed until third karyokinesis) and infectious *F. graminearum* multicellular macroconidia, the translucent, canoe-shaped asexual spores possessing 4–5 septa and derived from phialides, the conidium producing cells. Middle row (left): *T. thermophilus* undergoing cell division and demonstrating distinct localization of β-tubulin isotypes. Btu2 is enriched in somatic cilia (green lines on cell surface) and basal bodies (black dots beneath each cilium on cell surface; not all cilia are shown). Blt1 and Blt4 construct the spindle (red lines) in the mitotic division of the micronucleus (smaller blue circle) and also assemble MTs (red lines) during amitotic division of the macronucleus (large blue circle). Middle row (right): *C. reinhardtii* in interphase, showcasing diverse MT organelles including the two apical flagella (thick green lines projecting outward) and 4 MT rootlets (thinner green and light green lines emerging from basal bodies within the cell), which contains stable, acetylated α-tubulin. The mother (red) and daughter (dark red) basal bodies, several nucleus-basal body connectors (black lines), eyespot (brown) and chloroplast (grey object at cell posterior) are also shown. Bottom row (left): *P. polycephalum* depicted in respective amoeba and flagellate stages. The uninucleate amoeba (left) have unorganized MTs (green lines), randomly positioned nucleus (blue circle) with filopods (blue lines extending from the cytoplasm) that contributes to multidirectional movements. It reversibly transitions to the uninucleate, comma-shaped, flagellated, sexual spore myxamoeba (right). This phase demonstrates an anterior and posterior flagellum (thick green lines; anterior longer than posterior) that emanates from the basal body (red) as well as a flagellar cone of MTs (thinner green lines in conical arrangement, emanating from the apical basal body) that can extend to the dorsal side of the organism. The beak-shaped nucleus (blue) is positioned underneath the cone. Bottom row (right): *T. gondii* tachyzoite demonstrates a diverse array of complex MT-organelles. The spindle MTs (green lines) and the corset of 22 subpellicular MTs respectively nucleate from the centrioles (orange cylinders above the blue nucleus) and the Apical Polar Ring (APR) MTOC (orange circle at apical part of the cell). Also shown are the tubulin-based hollow cylindrical conoid (green cylinder above the APR), two preconoidal rings (grey circles) above it and two intraconoid MTs (green lines) within its circumference.

During budding yeast mitosis, the spindle must be positioned across the bud neck (Figure 2), which is accomplished by two major and functionally redundant mechanisms (McNally, 2013). Prior to anaphase, the Kar9 mechanism links MT plus ends to myosin-dependent transport along polarized actin cables to position the spindle at the bud neck. During anaphase, the Dyn1 mechanism utilizes bud membrane-localized dynein movement along astral MTs to pull one spindle pole into the bud. Cells can tolerate the loss of either mechanism, but complete loss of both is lethal (Miller et al., 1998). While strong overexpression of *TUB3* as the sole α-tubulin makes cells significantly sick, and additional removal of Kar9 further decreases fitness, *dyn1Δ* reduces fitness comparably more than *kar9Δ*, suggesting the Kar9 spindle positioning mechanism may be preferentially compromised in these cells (Denarier et al., 2021). It will be interesting to learn the comparable phenotypes of cells overexpressing Tub1 as their sole source of α-tubulin.

Although *tub3∆* cells survive with their α-tubulin coming only from the *TUB1* locus, they do not survive if the *TUB3* ORF is moved into the *TUB1* locus, under regulation of the endogenous *TUB1* regulatory elements (Nsamba et al., 2021). Conversely, while *TUB3* cannot support viability in *tub1∆* cells, viability is restored when the *TUB1* ORF is placed in the *TUB3* locus, under regulation of the *TUB3* regulatory elements. These ORF swapping results suggest that Tub1 likely can support one or more essential processes at lower concentrations than Tub3 is able. Placing the ORF of either α-tubulin isotype into both loci created cells expressing near wildtype levels of exclusively Tub1 or Tub3. Unlike cells strongly overexpressing Tub3 (Denarier et al., 2021), both Tub1-only and Tub3-only strains expressing normal overall α-tubulin levels grow at wildtype rates (Nsamba et al., 2021). While *tub3∆* cells are benomyl supersensitive, restoring proper α-tubulin levels with extra Tub1 produces more resistance than in wildtype cells (Schatz et al., 1986b). In contrast, Tub3-only cells expressing proper overall α-tubulin levels are highly sensitive (Nsamba et al., 2021). These opposite sensitivities suggest that, like *in vitro* (Bode et al., 2003), microtubules polymerized from either α-tubulin isotype have different functional properties in cells. Indeed, the Dyn1-mediated mechanism is more efficient on Tub1-only microtubules, while the Kar9 mechanism works better using Tub3 (Nsamba et al., 2021). Because each mechanism is optimized by a particular isotype, the microtubules containing both Tub1 and Tub3 in wildtype cells can sufficiently perform both processes. These results demonstrate that the differences in isotype properties can be leveraged to optimize the molecular mechanisms required to achieve diverse microtubule-dependent processes. Consistent with this idea, half of the ∼60 synthetic genetic interactions each displayed by Tub1 or Tub3 are in common, while the other half are unique to either isotype, suggesting they differentially mediate additional functions beyond spindle positioning (Nsamba et al., 2021).

Altogether, pioneering work in budding yeast revealed a high level of redundancy between tubulin isotypes, although with some detectable influence on cellular phenotypes. Continued efforts and technical advances have more recently demonstrated that these isotype-specific properties play a significant role in differentially mediating various MT-dependent cellular processes. The budding yeast model is well positioned for further elucidation of the molecular mechanisms of tubulin isotypes.

### Schizosaccharomyces pombe

Following the groundbreaking studies in budding and fission yeast that uncovered the cell-division cycle (CDC) genes as temperature sensitive mutants (Hartwell et al., 1973; Nurse, 1975), the fission yeast β-tubulin [*NDA3*; (Hiraoka et al., 1984)] and one of two α-tubulins [*NDA2*; (Toda et al., 1984)] were identified as cold sensitive nuclear division arrested mutants (Table 1) (Toda et al., 1983). Hybridization assays using the newly discovered *Chlamydomonas* α-tubulin cDNA (Silflow and Rosenbaum, 1981) as a probe soon revealed the second, and full complement of α-tubulins in fission yeast [*ATB2*; (Adachi et al., 1986)].

Similar to the situation in budding yeast, the α-tubulin genes in fission yeast are not equivalent in their ability to support viability. Disruption of *NDA2* renders cells inviable, while cells lacking *ATB2* continue to grow at normal rates in rich media but are hypersensitive to the microtubule destabilizer thiobendazole (Adachi et al., 1986). Like Tub3 in budding yeast, increased expression of *ATB2* can rescue cell viability in the absence of *NDA2* (Toda et al., 1984). Although *NDA2* mRNA level was observed to be significantly higher than *ATB2* transcript, at the protein level the two isotypes were observed to be equivalent (Adachi et al., 1986). Consistent with this, equivalent amounts Nda2-and Atb2-containing heterodimers are present in purified tubulin (Drummond et al., 2011). It should be noted however, that studies have also reported higher Nda2 relative to Atb2 by western blot (Radcliffe et al., 1998). Moreover, increased expression of *ATB2* mRNA induces a decrease in *NDA2* transcript level yet the inverse, increased *NDA2* expression does not cause decreased *ATB2* transcript (Adachi et al., 1986). Again, like what may occur in budding yeast, these latter two findings raise the possibility that the isotype ratio may be regulated under various culture conditions or cell cycle stages.

Despite their ability to support viability, there are indications that *NDA2* and *ATB2* are functionally distinct. Both isotypes are present at equivalent levels, at least under some conditions (Adachi et al., 1986), yet only one supports viability without increased expression (Toda et al., 1984; Adachi et al., 1986). While *ATB2* is non-essential, temperature sensitive alleles can disrupt processes including the spindle assembly checkpoint and nuclear movement (Radcliffe et al., 1998). They are also lethal in combination with cold sensitive *NDA2* alleles at permissive temperature, demonstrating *ATB2* is capable of influencing microtubule functions (Radcliffe et al., 1998). Moreover, as seen with budding yeast α-tubulin isoforms (Bode et al., 2003), Nda2 and Atb2 display distinct effects on microtubule dynamics *in vitro*. Microtubules assembled from only Nda2-containing heterodimers have slower apparent rate constants for MT assembly, slower growth rate and lower critical concentration of polymerization compared to polymers containing both Nda2 and Atb2 (Hussmann et al., 2016). Similar comparisons *in vivo* remain to be done.

The parallels observed thus far between α-tubulin isotypes in *S. cerevisiae* and *S. pombe* are striking. With the recent discoveries of how Tub1 and Tub3 differ functionally (Denarier et al., 2021; Nsamba et al., 2021), the stage is now set to investigate whether Nda2 and Atb2 differentially mediate microtubule functions as well. Like budding yeast, fission yeast is a preeminent model for the study of MAPs and regulatory factors controlling microtubule dynamics, organization, and function. Notably, how microtubules are used to support cell polarity and center the mitotic spindle in *S. pombe*, in contrast to positioning the spindle across the bud neck in *S. cerevisiae* (Figure 2), will likely reveal additional mechanisms by which tubulin isotypes contribute to diverse biological processes.

### Tetrahymena thermophila

*T. thermophila* is a unicellular, ciliated, protozoan that offers multiple advantages for studying tubulin isotypes. It uses four α- and eight β-tubulin isotypes to assemble a diverse set of spatiotemporally distinct microtubule-containing structures (Figure 2) (Suprenant et al., 1985; Gaertig et al., 1993; Gaertig, 2000) involved in processes including ciliary motility, cell division, conjugation, cell shape maintenance and oral apparatus organization (Table 1) (Williams, 1975). Notably, some of these functions are absent in other common microbial systems like yeast.

The first *Tetrahymena* α-tubulin gene, *ATU1*, was found by probing digested macronuclear DNA with heterologous α-tubulin probes from chicken, *Drosophila*, and *Chlamydomonas* (Callahan et al., 1984). Within a year, seven distinct isoelectric variants of tubulin polypeptides were identified: five α and two β although it was unclear whether each represented a gene or PTM (Suprenant et al., 1985). Of the five α-tubulin variants, three are present exclusively in ciliary, and the other two exclusively in cytoplasmic microtubules. While ciliary microtubules contain both β variants, cytoplasmic microtubules possess only a single variety. Two β-tubulin genes, *BTU1* and *BTU2,* were subsequently identified but found to encode identical polypeptides (Gaertig et al., 1993). Deletion of the α-subunit, *ATU1* (Hai et al., 1999), or simultaneous loss of both β-tubulin genes (Xia et al., 2000) is lethal. The idea that a single α- and β-tubulin polypeptide generated multiple isoelectric variants and supported the diverse array of structures in *Tetrahymena* focused attention on posttranslational modifications. These efforts revealed that α-tubulin in ciliary MTs is acetylated on the highly conserved K40 (Piperno and Fuller, 1985). This PTM is non-essential in *Tetrahymena* (Gaertig et al., 1995) and initially purported to improve Kinesin-1 binding and motility (Reed et al., 2006), but later reported that this effect may be indirect (Walter et al., 2012). Similarly, glutamylation was discovered (Bré et al., 1994) and later found to be non-essential but important for assembly and function of certain organelles such as the basal body (Wloga et al., 2008) as well as force generation by ciliary dynein (Suryavanshi et al., 2010). While both α- and β-tubulins are polyglycylated, only the α-tubulin modification was found to be dispensable (Xia et al., 2000; Thazhath et al., 2002). The majority of α-tubulin isotypes also undergo detyrosination (Redeker et al., 2005). Altogether these studies demonstrate the role of PTMs in mediating distinct MT functions and support their role in the multi-tubulin hypothesis.

The importance of the C-terminal tails of α- and β-tubulins was also investigated and both were found to be essential. Interestingly, while the absence of a tail on either α- or β-tubulin is not tolerated, the tails are interchangeable in that cells grow normally with either an α-or β-tail on both subunits (Duan and Gorovsky, 2002). Moreover, a modified α-tail supports viability when paired with a β-tail lacking posttranslational modification sites but not with a deletion of the β-tail, suggesting the tails themselves have essential roles independent of PTMs (Duan and Gorovsky, 2002). During this period, it was also recognized that when cells are deciliated, transcription of the α-tubulin, *ATU1*, and both β-isotypes, *BTU1* and *BTU2*, is induced, but when cytoplasmic microtubules are perturbed only *ATU1* and *BTU1* are induced (Gu et al., 1995). These data were amongst the earliest demonstrations of tubulin gene/isotype-specific response in cells and perhaps, unsuspectingly, foretold the isotype-localization results from *Tetrahymena* that would strongly support the multi-tubulin hypothesis (Pucciarelli et al., 2012).

Our understanding of tubulin isotype function in *Tetrahymena* changed dramatically when shotgun sequencing of the 104 Mb macronuclear genome revealed three additional α-tubulin and six more β-tubulin genes, named α-like tubulin (*ALT*) and β-like tubulin (*BLT*), respectively (Eisen et al., 2006). The *ALT*’s and *BLT*’s lack the characteristic polyglutamylation and polyglycylation PTM motifs on their CTTs that otherwise serve essential roles for their canonical counterparts. The discovery of multiple α- and β-tubulin isotypes further illuminated how this single celled organism assembles and controls diverse microtubule structures. Finally, a groundbreaking study demonstrated unique utilization of specific β-isotypes (Pucciarelli et al., 2012). Using live cell imaging of GFP-tagged tubulins and biochemical fractionation assays, Btu2 was shown to be highly enriched in somatic cilia and basal bodies, while Blt1 and Blt4 appear to be absent. Moreover, the latter two, but not Btu2 are used to assemble microtubules in the macronucleus as well as mitotic spindle of the micronucleus during cell division. During conjugation, Blt1 is the only of the three isotypes to build the micronuclear meiotic spindle. *Tetrahymena* is positioned as a strong model to further elucidate the molecular mechanisms of tubulin isotypes and PTMs in governing MT functions.

### Aspergillus nidulans

The filamentous ascomycote *A. nidulans* (Figure 2) served as an impressive pioneering model for the initial study of tubulins. Indeed, screens for sensitivity to benzimidazole, a MT destabilizer, initially uncovered *BENA* as the first known β-tubulin gene in any organism (Sheir-Neiss et al., 1978). Revertants of *ts-benA* mutants were leveraged to reveal *TUBA* (Morris et al., 1979), and two-dimensional electrophoresis using mutants of *BENA* or *TUBA* revealed the presence of at least one additional α- and β-tubulin gene (Morris et al., 1984). Its two α-tubulins, *TUBA* and *TUBB*, and two β-tubulins, *BENA* and *TUBC*, (Morris et al., 1984), were subsequently confirmed by cloning (Table 1) (Sheir-Neiss et al., 1978; Morris et al., 1979, Morris et al., 1984; Gambino et al., 1984; May et al., 1987; Doshi et al., 1991).

Notably, the α- and β-tubulin isotypes display more variations than is typical within a species, with ∼28 (Doshi et al., 1991) and ∼18% (May et al., 1985; May et al., 1987) sequence divergence, respectively. Disruption of either the α-tubulin isotype *TUBA* or *TUBB* disrupts karyokinesis or results in abnormal cell/nucleus morphology, respectively (Doshi et al., 1991). They also operate distinctly in specific stages of the life cycle. While *TUBA* is essential during vegetative growth (Oakley et al., 1987; Doshi et al., 1991), *TUBB* is essential for development before the first meiotic division (Kirk and Morris, 1991). However, overexpression of either α-tubulin isotype largely rescues the deleterious effects from loss of the other (Kirk and Morris, 1993). Thus, although the two α-tubulins possess significant redundancy, the data suggest they harbor at least some function difference.

Disruption of either β-tubulin also produces differing phenotypes. *BENA* is essential for viability and required during vegetative growth for nuclear migration and nuclear division (Oakley and Morris, 1980, Oakley and Morris, 1981; Oakley and Rinehart, 1985). On the other hand, although *TUBC* expression specifically increases during conidiation, the process of forming and reproducing via spores without conjugation (May and Morris, 1988), and affects MT function during this process (May et al., 1985), it is not essential during any part of the life cycle (May et al., 1985; Weatherbee et al., 1985). Moreover, placing *TUBC* behind the *BENA* promoter rescues the lethality of *BENA* loss, demonstrating the two isotypes have high redundancy (May, 1989). The resulting hypersensitivity to the MT destabilizer benomyl, however, reveals that like their α-tubulin counterparts, *TUBC* and *BENA* have at least some functional difference (May, 1989). Consistent with observations in other organisms, the results reveal that tubulin isotypes in *A. nidulans* share a high level of redundancy yet suggest they also impart unique aspects to MT function.

### Fusarium graminearum

*F. graminearum*, the anamorph of the notorious filamentous ascomycete, *Gibberella zeae*, is known for causing the economically devastating fusarium head blight (Figure 2) [reviewed in (Sutton, 2009; Leplat et al., 2013)]. Like *A. nidulans*, this fungus has two β-tubulin isotypes *FgTUB1* and *FgTUB2* (Lu et al., 2000) along with two α-tubulin isotypes *FgTUB4* and *FgTUB5* (Table 1) (Zhao et al., 2014). Following the observation that mutations in single genes result in resistance to the benzimidazole fungicides (Yuan and Zhou, 2005), both β-tubulin genes were screened for related mutations. Interestingly, despite high sequence identity with benzimidazole-resistant tubulins from other filamentous fungi, *FgTUB1* failed to display any causative mutations for resistance (Chen et al., 2007). Conversely, *FgTUB2* readily generated benzimidazole-resistant mutations (Chen et al., 2009; Liu et al., 2013). Altogether these findings indicate that the two β-tubulins in *F. graminearum* likely have distinct functional roles in the cell, or possibly different benzimidazole-binding properties, and suggest that individual tubulin isotypes can have cell context-specific interactions and/or functions that cannot be bridged by alternative isotypes.

Consistent with distinct functions, phylogenetic analysis indicates the β-tubulin isotypes in *F. graminearum* are under divergent selection pressure (Zhao et al., 2014). As a result, there are major amino acid divergences between them in the regions of GTP binding, intradimer interface, and the MT surface which could manifest as differences in properties including MT dynamicity and MAP interactions (Tassan and LeGoff, 2004; Luo et al., 2014). Loss of *FgTUB2* causes more severe growth defects than *FgTUB1*, but since only the loss of *FgTUB1* results in sterile perithecia (spore-producing fruiting body), it alone is essential for ascosporogenesis, or spores resulting from sexual reproduction (Zhao et al., 2014). It was recently found that although both β-tubulins have similar localization in all life cycle stages and overlapping functions during vegetative growth, they differ in their ability to support mycotoxin production (Wang et al., 2019). Additionally, while the transcription and translation of *FgTub1* increases in response to *FgTUB2* deletion, the opposite is not true. These data indicate that the tubulin isotypes in *F. graminearum* make distinct contributions to MT-dependent processes.

### Chlamydomonas reinhardtii

*C. reinhardtii*, a unicellular, biflagellated, photosynthetic algae, is a proven model to study cell motility, cytoskeleton biology, and intraflagellar transport. Interestingly, despite harboring a subset of relatively complex MT structures (Figure 2), its pair of α-tubulin (*TUA1* and *TUA2*) and pair of β-tubulin (*TUB1* and *TUB2*) isotypes (Minami et al., 1981; Silflow and Rosenbaum, 1981; Brunke et al., 1982b) each encode identical protein products (Table 1) (Youngblom et al., 1984; James et al., 1993). The diversity of MT structures formed in *Chlamydomonas* from a single α- and β-tubulin polypeptide directed efforts toward PTMs. Very early in tubulin investigation the α-tubulin composition in *Chlamydomonas* was found to differ isoelectrically between axonemes and the cell body (Witman et al., 1972; Brunke et al., 1982a). The fact that *TUA1* and *TUA2* encode identical proteins, together with the finding that they are equally expressed (Witman et al., 1972; Brunke et al., 1982b; McKeithan et al., 1983), reinforced the finding that α-tubulin is differentially acetylated in the two compartments (L’Hernault and Rosenbaum, 1983, L’Hernault and Rosenbaum, 1985; McKeithan et al., 1983; Piperno and Fuller, 1985). This acetylation, which can be reversed during flagella resorption (L’Hernault and Rosenbaum, 1985), may also be important for proper photoreceptor localization (Mittelmeier et al., 2011). Within properly formed axonemes, polyglutamylation of α- and/or β-tubulin subunits regulates the function of inner-arm dyneins to control flagellar motility by modulating MT-dynein interactions (Kubo et al., 2010, Kubo et al., 2012). Together these data illustrate the importance of tubulin PTMs and MT regulators, and demonstrate that a single α- and β-tubulin isotype are capable of building a range of diverse MT structures.

Moreover, the ability to readily fractionate subcellular structures such as axonemes makes this an ideal model to study the effects of various PTMs and MAPs on the MTs within (Orbach and Howard, 2019). Because the genes for each pair of isotypes encode identical polypeptides, screening for mutants with specific effects has been challenging. To overcome this, strains harboring single deletions of each tubulin isotype alone, as well as *tua1*-*tub1* and *tua2*-*tub1* in combination, were recently created (Kato-Minoura et al., 2020). Notably, the growth rate among the mutants is comparable, as is flagellar length and rate of growth following deflagellation. These deletion strains exhibit differing sensitivities to MT disrupting compounds and serve as more tractable platforms for uncovering tubulin mutations further altering drug sensitivity (Kato-Minoura et al., 2020). Such single isotype strains could also facilitate the investigation of PTMs and other regulatory mechanisms in the function of MT structures found in *Chlamydomonas*.

### Physarum polycephalum

*P. polycephalum*, a myxomycete or acellular slime mold, is a protist that harbors a relatively large complement of tubulin isotypes: five α-tubulins [*ALTA*, *ALTB(N)*, *ALTB(E)*, *ALTC*, *ALTD*] (Burland et al., 1983; Schedl et al., 1984b; Krämmer et al., 1985; Singhofer-Wowra et al., 1986; Green et al., 1987; Monteiro and Cox, 1987a, Monteiro and Cox, 1987b) and three β-tubulins (*BETA*, *BETB*, *BETC*) (Table 1) (Burland et al., 1984; Singhofer-Wowra et al., 1986; Burland et al., 1988; Solnica-Krezel et al., 1988; Werenskiold et al., 1988). This pool of tubulin isotypes is used to support a range of MT-structures and functions in this simple protist. The isotypes are differentially expressed and incorporated into MT structures at different life cycle/ploidy phases (Figure 2) of this organism, namely- 1) the multinucleated, unicellular plasmodium (diploid), 2) the uninucleate, unicellular myxamoeba (haploid), 3) the motile flagellate stage (also haploid), and 4) sclerotium (a compact formation of haploid spores) (Dove and Rusch, 1980; Schedl et al., 1984b). This tubulin complement builds astral, spindle, centriole, and flagellar MTs in myxamoeba; but constructs only intranuclear, anastral mitotic spindles in the multinucleated cell stage known as coenocytic plasmodia, which lacks cytoplasmic MTs (Havercroft and Gull, 1983; Solnica-Krezel et al., 1991).

Expression of the α-tubulin isotypes is differentially regulated in various life stages of *Physarum*. Despite having only 1 MT organelle, the anastral mitotic spindle, in the coenocytic plasmodia stage, more tubulin isotypes were found to be definitively expressed in this stage [*ALTA*, *ALTB(N)*, *ALTB(E)*, *BETB*, *BETC*] than the amoeba-like myxamoeba stage (*ALTA*, *BETA*, *BETB*) (Burland et al., 1983; Roobol et al., 1984; Gerber et al., 2022). Although the α-tubulin population contains two isoelectric variants, one was found to originate via acetylation of AltA (Green and Dove, 1984; Blindt et al., 1986; Diggins and Dove, 1987; Sasse et al., 1987), leaving only one major α-isotype expressed in the myxamoeba phase (Singhofer-Wowra and Little, 1987). While *ALTB(E)* encodes one of the two major α-tubulin isoelectric variants in plasmodia, the remaining four α-tubulin genes, and particularly *ALTC* and *ALTD*, potentially encode the other isoelectric variants (Cunningham et al., 1993).The single α-tubulin expressed in myxamoeba was also found to be less prone to mutation (Singhofer-Wowra et al., 1986) than its more divergent and multiple plasmodial counterparts (Krämmer et al., 1985). This suggests that the tubulin mutational rate may be limited by the structural constraints of highly conserved MT structures like the axoneme, which is present in myxamoeba and not the plasmodial phase, but also supports the idea that utilizing multiple tubulin isotypes allows individual isotypes to more readily coevolve with specific MT-dependent processes. In plasmodia, *ALTA*, *ALTB(N)*, *ALTB(E)*, *BETB*, and *BETC* are synthesized coordinately with mitosis, but after spindle disassembly the synthesis of *BETC* is reduced significantly more than *BETB* (Schedl et al., 1984a). While all these isotypes are incorporated into the intranuclear mitotic spindle (Paul et al., 1987), it remains unknown whether the α-tubulin isotypes are functionally interchangeable or not (Green et al., 1987).

Of the three β-tubulin isotypes, *BETA* and *BETC* are exclusively expressed and incorporated into MT structures of the myxamoeba and plasmodia stages, respectively (Burland et al., 1988; Diggins-Gilicinski et al., 1989; Gerber et al., 2022). On the other hand, *BETB* is constitutively expressed during both the myxamoeba and plasmodia phases (Werenskiold et al., 1988) and incorporated into all MT structures (Paul et al., 1989). Consistent with its expression profile, mutation in *BETB* alone can adequately confer benzimidazole resistance to both plasmodia and myxamoeba (Burland et al., 1984). Interestingly, akin to tubulins in budding yeast that do not construct complex MT organelles, *Physarum* plasmodial *BETC*, which also constructs only one MT-structure, i.e., the anastral mitotic spindle, potentially has fewer functional constraints. (Solnica-Krezel et al., 1988; Solnica-Krezel et al., 1991).Indeed, *BETC* has the most divergent sequence relative to the other two β-tubulin isotypes owing to neutral drift and/or positive selection for operating in plasmodial spindles (Diggins-Gilicinski et al., 1989). This suggests the requirement to participate in multiple MT-mediated processes may select for a high level of redundancy among an organism’s isotypes. Although it is absent in myxamoeba, *BETC* is still found in the astral spindles produced in developing cells during the amoeboid-to-plasmodial transition (Solnica-Krezel et al., 1988). Therefore, whether *BETC* has a specific role(s) in anastral spindles remains obscure.

Overall, the *Physarum* model offers the opportunity to explore redundancies and functional differences of tubulin isotypes in morphologically, phenotypically and genetically compartmentalized life cycle stages. Its tubulin isotypes display unique expression profiles and function in distinct, isolatable life stages. While research into *Physarum* tubulin isotypes has not progressed recently, much more remains to be explored.

### Toxoplasma gondii

*T. gondii* is an obligate intracellular parasite capable of infecting nucleated cells and causing serious complications, including birth defects and blindness in human and animal hosts (Halonen and Weiss, 2013). Despite displaying a diverse array of MT structures (Figure 2) in various life cycle stages (Morrissette and Sibley, 2002; Hu et al., 2006), it was initially thought to contain just one α-tubulin and one β-tubulin gene (Nagel and Boothroyd, 1988). Relatively recently, genomic data (www.toxodb.org) revealed two additional α- and β-tubulin isotypes, bringing the total to three each (Table 1) (Hu et al., 2006). Additionally, both α- and β-subunits harbor extensive PTMs (Xiao et al., 2010). Proteomic and transcriptomic data indicate that all six isoforms are expressed in tachyzoites and oocyst stages, yet α3 alone appears to have low expression in both stages (Hu et al., 2006; Xiao et al., 2010). The only isoforms detected in the cortical MT array by mass spectrometry analysis were α1, β1 and β2 (Wang et al., 2021), indicating that they potentially are the major isoforms expressed and/or enriched in this subpopulation. While the three β-tubulin isotypes have very high conservation (∼97% amino acid identity), the α-tubulins are quite diverse (40%–68% identity) (Xiao et al., 2010; Wang et al., 2021), strongly implying they may fill specialized roles. Consistent with this idea, α2 specifically has four extra amino acids inserted into the H1-S2(N) loop, responsible for lateral dimer interactions. This modification may potentially impart increased flexibility to MTs containing α2 subunits (Morrissette, 2015). The CTT of α3 is extraordinarily long and less acidic than commonly seen for α-tubulins, implying it may mediate atypical interactions. Thus, α2 and α3 may potentially function in specialized structures such as conoid and flagellar axonemal MTs (Morrissette, 2015). Moreover, only mutations in α1 have demonstrated resistance to the protozoan-specific MT inhibitors dinitroanilines (Morrissette et al., 2004; Ma et al., 2007, Ma et al., 2010). These recent breakthroughs into the structural and functional differences of α-tubulin isoforms in *T. gondii* position this medically relevant parasite as a model for elucidating further mechanisms for how tubulin isotypes mediate diverse MT-based organelles.

## Conclusion

The MT cytoskeleton is an indispensable component of eukaryotic cells. The overall structure of MTs is highly conserved, as are their main building blocks, α/β-tubulin heterodimers. Despite this high conservation, MTs accomplish a wide array of processes in diverse cellular contexts. A central question is how MTs perform such a range of tasks, often concurrently in the same cell. One common aspect in most organisms is the evolution of multiple α- and β-tubulin isotypes. How do multiple isotypes contribute to the diversity of MT functions? How is transcriptional and/or translational regulation, as well as post-translational modification of specific isotypes used to control MT behavior? How do unicellular organisms with relatively simple MT cytoskeletons benefit from multiple tubulin isotypes? These and other questions remain largely unanswered.

Microorganisms were well-represented in the vanguard of the initial discovery and characterization of tubulin isotypes. Their tractability readily allowed gene knockouts and overexpression, and facilitated subsequent analysis of cell viability and phenotypes. Some microorganisms possess only a few isotypes and a simplified repertoire of MT-dependent events, which can facilitate a mechanistic understanding of isotype contribution to the underlying processes. Others harbor several isotypes and utilize a more diverse array of MT-based structures. This allows investigation of more complex and diverse organism-specific MT arrays, such as the perithecia, or fruiting body, formation and ascosporogenesis in *Fusarium*, or highly conserved structures such as axonemes in *Tetrahymena* and *Chlamydomonas*. Moreover, distinct MT structures and dynamic behaviors are integrated into cell cycle and developmental specific stages, such as the myxamoeba-specific flagella in *Physarum*.

The early work on tubulin isotypes in microorganisms revealed two major themes. The first is that there appears to be a significant amount of redundancy among the isotypes with regard to basic MT functions. This is evident in the work described above where organisms often remain viable following loss of single isotypes. Moreover, when loss of a particular isotype is lethal, viability is often restored by increased expression of the remaining isotype(s), suggesting that at sufficiently high concentrations either isotype can adequately perform all essential functions. The second theme that emerged from work across a range of organisms is that loss of individual isotypes, while often not lethal, nearly always results in some perturbation to cellular processes and/or phenotype. Together these results demonstrate that many individual isotypes are not essential, yet most may contribute one or more unique properties that facilitate overall MT function.

The generalized finding that individual isotypes are often not required for viability demonstrated that, at least in most cases, they do not exclusively mediate specific MT functions. Phenotypes resulting from the loss or overexpression of isotypes are also difficult to separate from potential indirect effects resulting from changes in overall tubulin level and/or stoichiometries of the remaining isotypes. These aspects, together with technical challenges with direct gene replacement and obtaining recombinant tubulins, likely contributed to a relative decrease in research efforts investigating the multi-tubulin hypothesis from the tubulin isotype perspective. Work in microorgansisms also demonstrated that some species assemble a diverse array of MT structures using only a single form of α- and β-tubulin subunits. This is most evident in *Chlamydomonas* cells, which construct structures including mitotic and meiotic spindles, apical MT rootlets, basal bodies and flagella. Although *Chlamydomonas* harbors two α- and two β-tubulin genes, they each encode identical polypeptide sequences. This demonstrates that utilizing multiple tubulin isotypes is not a fundamental requirement for a wide range of MT structures and functions, and that the effects of PTMs on MTs and their regulatory proteins are also a critical part of the multi-tubulin hypothesis, or “tubulin code.” Indeed, the finding that *Chlamydomonas* utilizes just one form of α- and β-tubulin heterodimer stoked research into the functions of PTMs in this and other microorganisms including *Tetrahymena*.

Continued work has shown that some isotypes do serve essential and exclusive roles in specialized structures such as axonemes in Drosophila sperm cells (Hoyle and Raff, 1990; Hutchens et al., 1997), or the highly curved ring-like MTs of the marginal band in mammalian blood platelets (Schwer et al., 2001). Moreover, recent studies using gene replacement to control for overall tubulin levels clearly show that the absence of individual isotype can impair specific aspects of MT function (Honda et al., 2017; Nishida et al., 2021; Nsamba et al., 2021). For instance, both α-tubulin isotypes in budding yeast can support viability, yet each is required to optimize the two distinct spindle positioning mechanisms (Nsamba et al., 2021). Similarly, several neuronal specific β-tubulin isotypes in mouse are not needed for viability (Latremoliere et al., 2018; Bittermann et al., 2019) but, for instance, Tubb3 is specifically required for the timely regeneration of peripheral nervous system axons following injury (Latremoliere et al., 2018).

Overall, a central role of tubulin isotypes, which is consistent with the multi-tubulin hypothesis, is to allow MTs to more effectively perform diverse functions. In most organisms the MT cytoskeleton must concurrently support multiple processes and interactions. Thus, a high level of redundancy among isotypes is, at least in part, constrained by the need for αβ-tubulin heterodimers to maintain the ability to copolymerize and undergo dynamic instability. Their structure and function are further constrained by the requirement to at least minimally support the activities of ubiquitous and central regulators and motor proteins. Beyond these constraints, tubulin isotypes are able to coeveolve with distinct sets of regulatory proteins within organisms, and among various branches of evolution. Another key aspect that has become clear from work in microorganisms, as well as in other model systems (Nsamba and Gupta, 2022), is that α- and β-tubulin tubulin isotypes are able to significantly influence the properties and function of cellular MTs when present at substochiometric levels within the polymer. This quality is critical for understanding how mutations in a single copy of various isotypes can underlie the range of observed tubulinopathies.

Since the early investigation of tubulin and microtubule function, microbial models have provided leading insights into the role of tubulin isotypes and allowed testing of the multi-tubulin hypothesis. In a diverse range of microorganisms the groundwork has been laid that demonstrates their tubulin isotypes differentially facilitate various MT functions. Although at a slower pace, studies in microorganisms continue to yield additional details of the multifaceted role of tubulin isotypes. As advances in genomics, proteomics, and genetic manipulation expand the tractability of microbial models, they will undoubtably continue to make valuable contributions toward elucidating the role of isotypes in fundamental MT functions. Studying tubulin isotypes in a range of organisms will also reveal how they contribute to the diversity of cytoskeletal function seen across biology.
